# Influencing factors of colonoscopy screening in first-degree relatives of hospitalized colorectal cancer patients and preliminary clinical practices to improve the compliance

**DOI:** 10.3389/fonc.2025.1533475

**Published:** 2025-04-28

**Authors:** Dongqin Zhao, Fan He, Chen Luo, Huanhuan Huang, Qinghua Zhao

**Affiliations:** ^1^ Department of Nursing, The First Affiliated Hospital of Chongqing Medical University, Chongqing, China; ^2^ Department of Gastrointestinal Surgery, The First Affiliated Hospital of Chongqing Medical University, Chongqing, China; ^3^ Department of General Surgery, Xinhua Hospital, Shanghai Jiao Tong University School of Medicine, Shanghai, China

**Keywords:** colorectal cancer, first-degree relatives, screening, colonoscopy, compliance

## Abstract

**Objectives:**

This study aimed to analyze the factors that influence colonoscopy screening in first-degree relatives (FDRs) of patients with colorectal cancer (CRC) and explore the feasibility to invite FDRs to undergo a colonoscopy to improve screening compliance.

**Methods:**

Retrospective analysis based on a prospectively collected database of which FDRs of CRC patients who visited our center between April 2021 and October 2021 and received a questionnaire surgery. The questionnaire contained three aspects: demographic and lifestyle factors, health beliefs, and disease cognition. The FDRs were invited to undergo a colonoscopy and were followed-up by telephone regarding colonoscopy compliance one year later.

**Results:**

In total, 303 FDRs from 256 patients with CRC were analyzed. Among them, 113 underwent colonoscopy, with a colonoscopy compliance rate of 37.3%. The results of the multivariate analysis showed that the FDRs who underwent colonoscopy were older (OR=2.32, p=0.006), had commercial insurance (OR=2.23, p=0.013), had multiple family members with CRC (OR=3.04, p=0.012), had higher cognition of CRC (OR=3.02, p=0.006), had high self-efficacy for disease screening (OR=1.14, p=0.026), and accepted colonoscopy appointment sheet to undergo colonoscopy screening (OR=4.51, p<0.001), which were influencing factors for CRC screening in FDRs.

**Conclusion:**

This study found that FDRs who were ≥40 years old, had commercial insurance, had multiple family members with CRC, had higher cognition of CRC, had high self-efficacy for disease screening, and received a colonoscopy appointment while in the hospital were more willing to undergo colonoscopy screening. Studies could further validate the feasibility of this approach in the future.

## 1 Introduction

Colorectal cancer (CRC) ranks the third most common malignant tumor worldwide (accounting for 10.2% of new cancer cases), and is the second most common cause of cancer-related deaths (9.2% of cancer-related deaths) (1). Approximately half of these new cases and deaths occurred in Asia (2). In patients with early-stage (I, II stages) CRC, the 5-year survival rate reaches 87%~90%, while in stage III patients, it drops to 68%-72% and in stage IV patients, the 5-year survival rate is only 11%-14% (3). Tumor screening is the simplest way to diagnose cancer at an early stage (4), especially in high-risk individuals with a family history (5, 6).

Studies have shown that genetics may play a role in approximately 25-30% of CRC cases (7, 8). Individuals with a family history of CRC are at a higher risk of developing cancer, especially the first-degree relatives (FDRs) (including children, parents, and siblings) (9), who have a nearly twofold increased risk compared with the general population. Most published guidelines recommend early CRC screening in FDRs to avoid an adverse prognosis (10, 11). Colonoscopy is the most direct screening program for CRC, which can intuitively detect lesions and achieve therapeutic objectives at the same time (12).

The colonoscopy screening rate in adult FDRs worldwide is still unsatisfactory at approximately 26-45% (13). Moreover, the adult FDRs undergo colonoscopy screening compliance is lower in China, only 15-21% (14, 15). The proportion of young-onset CRC is increasing yearly, and due to the lack of screening, the majority of patients are diagnosed at advanced stages (16). Serife Koc et al (17) and Du et al (14) found that the participation of CRC screening in FDRs is related to health cognition, behaviors and beliefs. And Li et al (18) think that fear of CRC, economic status, and the healthcare insurance status also affect FDR screening. They all analyzed only a subset of the reasons that influenced to undergo colonoscopy screening by FDRs, and did not simultaneously explore targeted measures to increase their colonoscopy screening compliance. In China, FDRs who accompany CRC patients during hospitalization have access to a large amount of tumor-related knowledge, including the development of tumors, screening methods, treatment, and prognosis. However, although FDRs can receive many recommendations for CRC screening, no CRC screening program has been developed for FDRs of hospitalized CRC patients. The colorectal surgery ward is an ideal location for FDRs of CRC patients to CRC screening.

The purpose of this study was to analyze the influencing factors of colonoscopy screening in FDRs of inpatients with CRC and explore the feasibility to invite FDRs to undergo a colonoscopy to improve screening compliance.

## 2 Methods

### 2.1 Participants

The participants were FDRs (including children, parents and siblings) of patients with CRC who were admitted to the Department of Gastrointestinal Surgery at the First Affiliated Hospital of Chongqing Medical University between April 2021 and October 2021.

The inclusion criteria were: 1.The FDRs of patients diagnosed with CRC (The pathological diagnosis was adenocarcinoma); 2.FDRs aged >18 and <75 years; 3.FDRs were aware that the patient had CRC; 4.FDRs were able to complete the questionnaire and volunteered to participate in the research.

Exclusion criteria were: 1.FDRs had been diagnosed with CRC or other cancers; 2.FDRs were in poor general condition and were unable to undergo a colonoscopy.

This study was approved by the First Affiliated Hospital of Chongqing Medical University (Ethical ID: 2020-358) and was in accordance with the Declaration of Helsinki.

### 2.2 Study procedure and data collection

Prospective data on FDRs of patients were retrospectively analyzed. All FDRs signed informed consent forms. The questionnaire was provided to the FDRs after the surgeon communicated with the patients and their family members about the treatment plan. The questionnaire was accessed by scanning a QR code, and the researcher explained the content of the questionnaire to the FDRs using uniform instruction language, with each entry explained as consistently as possible without guidance. The time required to complete the questionnaire was greater than 3 minutes. The eligibility of the questionnaire was checked immediately after completion.

After submitting the questionnaire, FDRs who are older than 40 years of age or within 10 years of the onset of CRC in their family member will be asked if they wish to undergo colonoscopy screening. The doctor issued a colonoscopy appointment for FDRs who wished to undergo a colonoscopy. The colonoscopy appointment sheet issued for the FDRs was stamped with an exclusive seal and the sheet was valid for two years. In addition, the endoscopy center could open exclusive channels for participants in this screening to shorten the waiting time. One year after the completion of the questionnaire, the FDRs were followed up regarding colonoscopy screening compliance by telephone and the hospital electronic medical record system.

### 2.3 Instruments

The questionnaire was designed to collect FDRs’ data on demographic and lifestyle factors, health behaviors, health beliefs, and disease cognition. It contained the following 3 sections:

1. Demographic and lifestyle factors: The factors that might influence compliance with CRC screening were determined by reviewing the literature (19, 20). These factors included sex, age, body mass index (BMI), employment status (part-time or no job vs full time job), location of residence (rural or urban), marital status (single/divorced vs married), education level (primary school, junior high school, senior high school, college or bachelor’ degree and master degree or higher), family income (<2000, 2000-4000, 4001-6000, and >6000), and health insurance status (employee medical insurance vs resident or without medical insurance). Potential influencing factors such as, smoking (yes/no), alcohol consumption (yes/no), frequency of physical examinations (never, occasional and regular), willingness to free of charge screening (yes/no), history of chronic diarrhea (yes/no), and family history of CRC (1 vs >1) were also included. Participants who smoked at least one cigarette and drank one glass of alcohol a day were considered smokers or alcohol consumers. Individuals who participated in physical examination at least once a year were considered to have regular physical examination, who participated in physical examination only when they feel unwell were considered occasional physical examination, and who have not had physical examination were considered never to participate in physical examination.
2. Health beliefs: Information on the health beliefs of FDRs was investigated and collected using the Chinese version of CRC Health Belief Scale (HBS) which was translated by Wu et al (21) And the English version of CRCHBS was developed by Jacobs (22) based on Champion’s HBS. It not only reflects an individual’s cognition of a disease, but also reflect the response measures they will take in the face of diseases, which significantly affects compliance with disease screening (23). Health beliefs were evaluated by the following six aspects: 1. perceived susceptibility (five items); 2. perceived barriers to screening (six items); 3.perceived severity (seven items); 4.perceived benefits of screening (six items); 5. motivation for health (seven items); and 6. screening self-efficacy (five items). All items were rated on a 5-point scale ranging from 1 (completely disagree) to 5 (completely agree), except for items of perceived barriers, which were scored from 5 (completely disagree) to 1 (completely agree). The higher a participant’s HBS score, the higher their belief in healthy behavior (24). In the study by Ozsoy SA et al (25), the internal consistency of the subscales ranged from 0.54 to 0.88. In our study, we also showed good reliability and validity, with a Cronbach’s α coefficients of 0.854 for the total scale and 0.762 to 0.913 for the six aspects.
3. Health cognition and behavior: The scale for CRC screening by Hong et al. (26) from the Chinese University of Hong Kong was used and modified appropriately. We added to the health cognition section asking FDRs whether they were willing to participate in colonoscopy screening. To evaluate the CRC cognition and behavior of FDRs of the hospitalized patients, four main aspects were included: knowledge of CRC, risk factors associated with CRC, knowledge of CRC screening methods, and whether colonoscopy is the best screening strategy. The scale included 9 symptoms related to CRC, 12 disease-related factors, 6 main examination methods and colonoscopy as the main screening method. The mastery of CRC cognition was roughly divided into the following three levels: (1) a high level of cognition (known 4 factors); (2) a low level of cognition (known ≤ 2 factors); and (3) partial cognition (known 3 factors) (27). The questionnaire had good validity and reliability, with a Cronbach’s α coefficient of 0.801 and retest reliability of 0.758 (28), and the Cronbach’s α coefficient in our study was 0.840.

### 2.4 Statistical analysis

SPSS 27.0 (IBM Corp.; Armonk, NY, USA) was used for statistical analysis, and p-value < 0.05 was considered statistically significant. Mean and standard deviation (SD) were used to represent continuous variables, and Student’s t-test was used to compare the difference between groups. Frequencies and percentages were used to represent categorical variables, and Chi-squared or Fisher’s exact tests were used for group comparison. According to the results of the univariate analysis, the indicators with statistical significance (p < 0.05) for FDR colonoscopy screening were subject to multivariate logistic regression analysis. Uni- and multivariate analyses were performed to determine the independent influencing factors for colonoscopy screening compliance in the FDRs.

## 3 Results

**Figure 1:**
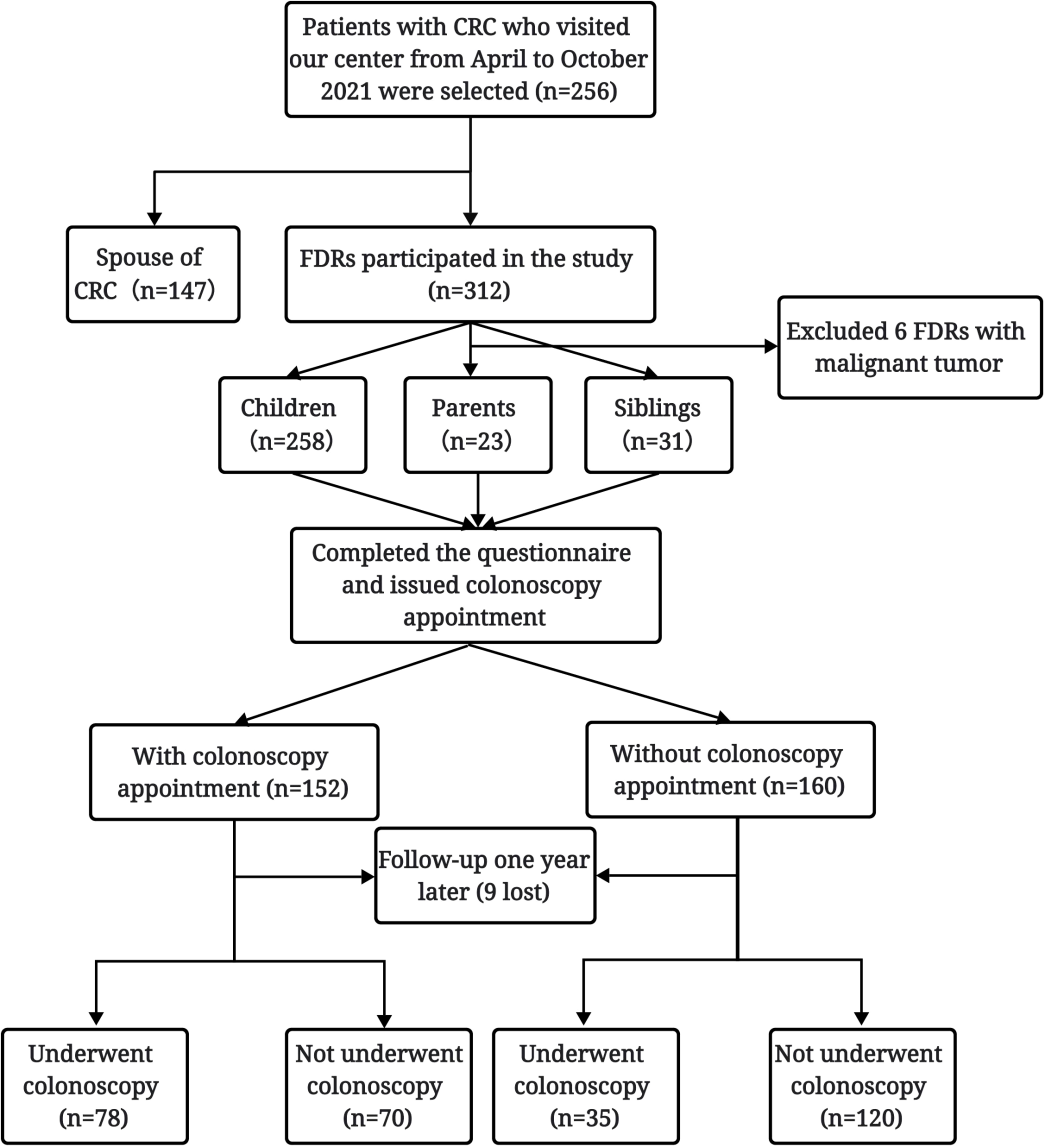
Flowchart of the study procedure.

A total of 318 FDRs of 256 CRC patients hospitalized in our center from April 2021 to October 2021 were initially investigated, among whom 6 FDRs with a history of malignant tumor were excluded. Finally, 258 children, 23 parents, and 31 siblings of patients with CRC were included in the analysis. Among the 312 FDRs, nine were lost to follow-up, and 303 completed follow-up one year after completing the questionnaire and were included in the study. Among these FDRs, 113 completed colonoscopy screening, with a colonoscopy screening compliance rate of 37.3% (**Figure 1**).

### 3.1 Demographic and lifestyle factors

More than half of the FDRs in this study were ≥ 40 years old (58.1%) and women (58.7%). Most of the participants (74.3%) lived in urban areas, and the vast majority (92.4%) had an education level of at least junior high school, and worked full-time (53.3%). However, less than half have employee or commercial health insurance (37.0%, 34.0%, respectively). In addition, a majority of respondents (83.2%) had a medical examination, 25.1% of FDRs had a history of chronic diarrhea, and 15.8% of FDRs had multiple FDRs with CRC in their family (**Supplementary Table 1**).

Compared with those who did not underwent a colonoscopy, the FDRs undergwent a colonoscopy were older (67.3% ≥ 40 years old, p < 0.001), had longer-term residence in urban area (n=92, 81.4%, p = 0.028), had commercial health insurance (43.3% vs 28.4%, p=0.008), underwent regular physical examinations (42.5% vs 23.2%, p = 0.002), had a history of chronic diarrhea (31.9% vs 21.1%, p = 0.036), were willing to undergo free of charge screening (86.7% vs 73.2%, p = 0.006), had multiple family members with CRC (23.9% vs 11.1%, p = 0.003), and had high compliance with colonoscopy screening. There were no significant differences in sex, BMI, employment status, marital status, education level, family income, smoking, and alcohol consumption (p > 0.05) (**Table 1**).

**Table 1:** Univariate logistic regression analysis of the impact of demographic and lifestyle factors on colonoscopy screening in FDRs of patients with CRC.

Characteristics	Underwent colonoscopy		p value
	Yes (n=113)	No (n=190)	
Sex			0.175
Male	41(36.3%)	84(44.2%)	
Female	72(63.7%)	106(55.8%)	
Age(years)			0.001*
<40	37(32.7%)	89(46.8%)	
≥ 40	76(67.3%)	101(53.2%)	
**BMI (kg/m^2^)**	22.61 ± 3.29	23.06 ± 3.58	0.280
Employment status			0.469
Part-time or without job	56(49.6%)	86(45.3%)	
Full-time job	57(50.4%)	104(54.7%)	
Residence location			0.028*
Rural	21(18.6%)	57(30.0%)	
Urban	92(81.4)	133(70.0%)	
Marital status			0.836
Single or divorced	18(15.9%)	32(16.8%)	
Married	95(84.1%)	158(83.2%)	
Educational level			0.473
Primary school	8(7.1%)	15(7.9%)	
Junior high school	25(22.1%)	60(31.6%)	
Senior high school	30(26.5%)	42(22.1%)	
College/bachelor’s degree	45(39.8%)	65(34.2%)	
Graduate degree or higher	5(4.4%)	8(4.2%)	
Family income (yuan)			
< 2000	18(15.9%)	40(21.1%)	0.086
2000–4000	24(21.2%)	59(31.1%)	
4001–6000	49(43.4%)	60(31.6%)	
> 6000	22(19.5%)	31(16.3%)	
Smoking			0.404
No	82(72.6%)	146(76.9%)	
Yes	31(27.4%)	44(23.1%)	
Alcohol consumption			0.117
No	87(77.0%)	160(84.2%)	
Yes	26(23.0%)	30(15.8%)	
Health insurance status			0.241
Employee medical insurance	37(32.7%)	75(39.5%)	
Resident or without medical insurance	76(67.3%)	115(60.5%)	
Commercial health insurance			0.008*
No	64(56.7%)	136(71.6%)	
Yes	49(43.3%)	54(28.4%)	
Frequency of physical examination			0.002*
Never	16(14.2%)	35(18.4%)	
Occasional	49(43.4%)	111(58.4%)	
Regular	48(42.5%)	44(23.2%)	
History of chronic diarrhea			0.036*
No	77(68.1%)	150(78.9%)	
Yes	36(31.9%)	40(21.1%)	
Willingness to free-finance screening			0.006*
No	15(13.3%)	51(26.8%)	
Yes	98(86.7%)	139(73.2%)	
Family history of CRC			0.003*
1	86(76.1%)	169(88.9%)	
> 1	27(23.9%)	21(11.1%)	

FDRs, first-degree relatives; BMI, body mass index; CRC, colorectal cancer; * p<0.05.

### 3.2 Health cognition and beliefs

The FDRs included in the study had high HBS scores (124.77 ± 23.37). Only a small number of patients were unaware of the symptoms (n=45, 14.9%), risk factors (n=57, 18.8%), and the screening methods (n=13, 4.3%) of CRC. A majority of FDRs (n=229, 75.6%) knew that colonoscopy was the most effective screening method for CRC. More than half of the FDRs had a high level of cognition in CRC patients (n=181, 59.7%) (**Table 2**).

**Table 2:** HBM score and knowledge of CRC in FDRs of patients with CRC.

Items	Mean ± SD/n(%)
Health beliefs	
**HBM score**	124.77 ± 23.37
Susceptibility	14.31 ± 4.99
Barriers	20.05 ± 5.47
Severity	23.58 ± 5.62
Benefit	23.08 ± 5.56
Impetus	26.49 ± 4.63
Efficacy	17.72 ± 4.43
Health cognition and behaviors	
Knowledge of CRC	
Knew all of the symptoms	12 (4.0%)
Knew none of the symptoms	45 (14.9%)
Knew all of the risk factors	3 (1.0%)
Knew none of the risk factors	57 (18.8%)
Knowledge of cancer screening	
Knew all of the screening methods	70 (23.1%)
Knew none of the screening methods	13 (4.3%)
Knew colonoscopy	229(75.6%)
Cognition of CRC	
Low level	74(24.4%)
Middle level	48(15.8%)
High level	181(59.7%)

HBM, Health Belief Model; CRC, colorectal cancer; SD, standard deviation.

The HBS scores of FDRs who underwent colonoscopy were significantly higher than those of FDRs who did not undergo colonoscopy (133.80 ± 13.95 vs. 120.19 ± 15.67, p < 0.001), and the scores regarding susceptibility (15.27 ± 5.08 vs. 13.73 ± 4.85, p = 0.009), the benefit of screening (25.01 ± 4.78 vs. 21.93 ± 5.68, p < 0.001), the barriers to screening (21.90 ± 5.02 vs. 19.13 ± 5.50, p < 0.001), motivation for screening (28.02 ± 3.98 vs. 25.58 ± 4.76, p < 0.001) and self-efficacy for screening (19.46 ± 3.98 vs. 16.69 ± 4.37, p < 0.001) were significantly different. There was no significant difference in CRC severity (p = 0.066). Colonoscopy compliance was significantly higher in FDRs with high levels of cognition of CRC than in those with moderate or low levels of cognition (75.2% vs. 50.5%, p < 0.001) (**Table 3**).

**Table 3:** Univariate logistic regression analysis of the association of the HBM score, cognition of CRC and colonoscopy appointment sheet with colonoscopy screening in FDRs of CRC patients.

Dimension	Underwent colonoscopy		p value
	YES (n=113)	NO (n=190)	
HBM score	133.8 ± 13.95	120.19 ± 15.67	<0.001*
Susceptibility	15.27 ± 5.08	13.73 ± 4.85	0.009*
Severity	24.35 ± 5.92	23.13 ± 5.39	0.066
Barriers	21.90 ± 5.02	19.13 ± 5.50	<0.001*
Benefit	25.01 ± 4.78	21.93 ± 5.68	<0.001*
Impetus	28.02 ± 3.98	25.58 ± 4.76	<0.001*
Efficacy	19.46 ± 3.98	16.69 ± 4.37	<0.001*
Cognition of CRC			<0.001*
Low level	17(15.0%)	57(30.0%)	
Middle level	11(9.7%)	37(19.5%)	
High level	85(75.2%)	96(50.5%)	
Colonoscopy appointment sheet			<0.001*
No	35(31.0%)	120(63.2%)	
Yes	78(69.0%)	70(36.8%)	

FDRs, first-degree relatives; CRC, colorectal cancer; HBM, health belief model; * p<0.05.

### 3.3 Colonoscopy appointment

Results showed that female, ≥ 40 years old, with higher family income, with a history of chronic diarrhea, and family members with multiple FDRs with CRC were more likely to accept a colonoscopy appointment (**Supplementary Table 2**). Among the FDRs who were participated in colonoscopy screening, accepted the colonoscopy appointment sheets significantly increased FDR compliance during the one-year follow-up period (69.0% vs. 36.8%, p < 0.001) (**Table 3**). The results showed that more FDRs were screened by colonoscopy through the exclusive screening channel when colonoscopy appointment sheets were received (n=45,30.4% vs. n=6,3.9%, p < 0.001). There was no significant difference in whether the FDRs underwent colonoscopy screening in other hospitals (n=33, 22.4% vs. n=18.7%, p = 0.439) (**Supplementary Table 3**).

### 3.4 Multivariate logistic regression analysis

The multivariate analysis found that colonoscopy screening compliance in FDRs aged ≥ 40 years was 2.3 times that in those aged < 40 years (OR = 2.3, p = 0.006). Colonoscopy compliance in FDRs with commercial health insurance was 2.2 times higher than that in FDRs without commercial health insurance (OR = 2.2, p = 0.013). FDRs with multiple family members with CRC were 3.0 times more likely to comply with colonoscopy screening than those with a single family member (OR = 3.0, p = 0.012). With each single-unit increase in disease screening efficacy, the probability of FDRs participating in CRC screening was 1.14 times higher than the probability of not participating. (OR = 1.14, p = 0.026). The colonoscopy screening compliance rate in FDRs with a high cognitive level of CRC was 2.0 times that in FDRs with a low cognitive level (OR=3.0, p = 0.006). In addition, colonoscopy compliance was 3.5 times higher in FDRs who accepted to get a colonoscopy appointment sheet (OR=4.5, p < 0.001) (**Table 4**).

**Table 4:** Multivariate logistic regression analysis of the factors associated with colonoscopy screening in FDRs of CRC patients.

Factor category	β	OR	95% CI for EXP(B)		p value
			Lower	Upper	
**Age (≥ 40 vs < 40 years old)**	0.843	2.322	1.274	4.234	0.006*
**Residence location (urban vs rural)**	-0.283	0.754	0.344	1.650	0.479
**Commercial medical insurance (yes)**	0.800	2.226	1.180	4.199	0.013*
Frequency of physical examination					
Never	Reference				
Occasional	-0.353	0.702	0.288	1.713	0.437
Regular	0.723	2.061	0.799	5.319	0.135
**History of chronic diarrhea (yes)**	0.626	1.870	0.948	3.686	0.071
**Willingness to free-finance screening (yes)**	0.513	1.670	0.761	3.661	0.201
**Multiple family history of CRC (1 vs 1)**	1.111	3.036	1.282	7.194	0.012*
**Colonoscopy appointment sheet (yes)**	1.550	4.506	1.421	9.571	< 0.001*
HBM					
Susceptibility	-0.039	0.962	0.900	1.028	0.251
Benefit	-0.004	0.996	0.920	1.078	0.915
Barriers	-0.053	0.949	0.873	1.031	0.214
Impetus	0.017	1.017	0.921	1.123	0.734
Efficacy	0.132	1.141	1.016	1.281	0.026*
Cognition of CRC					
**Low level**	Reference				
Middle level	-0.038	0.962	0.343	2.702	0.942
High level	1.106	3.021	1.381	6.611	0.006*

FDRs, first-degree relatives; CRC, colorectal cancer; OR, odds ratio; CI, confidence interval; HBM, HBM, Health Belief Model; CI, confidence interval; CRC, colorectal cancer; * p < 0.05.

## 4 Discussion

This study investigated the impact of demographic and lifestyle factors, disease cognition, health beliefs and health behaviors of FDRs on the colonoscopy screening compliance. We found that being over 40 years old, having commercial insurance, having multiple family members with CRC, having a high level of CRC cognition, and having high self-efficacy for disease screening were independent influencing factors of colonoscopy screening. Besides, the FDRs who accepted a colonoscopy appointment sheet were more likely to undergo colonoscopy screened. The colonoscopy screening compliance of FDRs in this study was 37.3%, higher than the screening rate of 18.9% (14), the FDRs of hereditary CRC screening rate of 23.0% (27), and the high-risk population of 14.0% (29) in other study.

### 4.1 Impact of demographic and lifestyle on colonoscopy screening

The results showed that compliance with colonoscopy was significantly higher in older FDRs (≥40 years) than in younger FDRs (<40 years). The American Multi-Social Task Force recommends that FDRs with a high risk of CRC should begin colonoscopy screening at 40 years of age or 10 years before the occurrence of CRC in the youngest family member and should be screened every 5 years (30). In addition, most staff in our center often recommend colonoscopy screening after 40 years of age when providing health education to patients, relatives, or healthy people. However, most young CRC patients are diagnosed at an advanced stage (16). Therefore, the screening behavior of young FDRs should be strengthened.

In addition, the colonoscopy screening rate of FDRs with commercial insurance was 43.3%, which was significantly higher than that of 28.4% in those without commercial insurance. This may be because the family conditions of individuals with commercial insurance are better, and they are more health-conscious and more willing to undergo colonoscopy screening. This is consistent with data from the U.S. National Survey, which found that among the general population of the United States, the colonoscopy compliance rate was 39% among individuals with health insurance and only 19% among those without health insurance (31). Nevertheless, in clinical practice, medical staff should strengthen health education for people without commercial insurance and increase their health cognition to improve their compliance with CRC screening.

FDRs with multiple family members had a higher compliance with colonoscopy screening. Although the majority of CRC cases are sporadic, 25% of the patients have a familial predisposition (32). Enrique et al. believed that FDRs with two family members with CRC showed a significantly increased risk of advanced tumors (33). Samadder et al (9) reported that CRC shows familial aggregation and usually develops in multiple members of a family, suggesting that in future studies, family based screening programs can be implemented to improve CRC screening compliance (34).

### 4.2 Impact of health beliefs on colonoscopy screening

Studies (35) have shown that people with higher health beliefs tend to take more measures to screen and treat diseases. The HBM can effectively reflect the subjects’ enthusiasm and compliance with disease screening. Our results showed that there was no significant association between the compliance of FDRs with colonoscopy screening and the perceived CRC susceptibility and severity scores in the ward; the higher an individual’s self-efficacy for cancer screening, the higher their compliance with colonoscopy screening.

The univariate analysis found that susceptibility, screening benefits, screening barriers, screening motivation, and screening efficacy had significant effects on FDR participate in colonoscopy screening. However, in multivariate analysis combine with demography data and FDRs’ knowledge of CRC, we found only that high efficacy of disease screening was an influential factor for FDRs to participate in colonoscopy screening. This is at odds with the conclusions of Du and serifi’s study. Du et al (14) identified health motivation as the strongest predictor of participation in colonoscopy screening by FDRs, and Serifi et al (17) identified perceived susceptibility and perceived barriers as the most important predictors. On the one hand, the susceptibility and severity of the disease in FDRs were related to the patients’ lifestyle and cognition of CRC. On the other hand, in the HBM (22), the main content used to evaluate the self-efficacy of FDRs to undergo CRC screening is whether they can recognize early symptoms of CRC and how to screen for CRC. These contents are often mentioned in our clinical work for patients and their families and are also introduced in the disease popular science exhibition in the ward. Therefore, most FDRs who undergo colorectal screening during companion can learn more about the pathogenesis, diagnosis, treatment, and prognosis of CRC. This may explain why the self-efficacy of FDRs for CRC screening was higher in those who underwent a colonoscopy in this study.

### 4.3 Impact of health cognition on colonoscopy screening

In this study, 59.7% of the FDRs had a high level of cognition of CRC. Colonoscopy compliance of FDRs with a high level of cognition was significantly higher than that of FDRs with moderate and low levels of cognition. Jones et al. and Ghanouni et al. believe that the higher an individual’s awareness of a disease, the better they can perceive the danger when the disease comes and properly deal with it (36, 37). They also had a better understanding of disease screening and were more willing to participate in early disease screening. However, 24.4% of the FDRs in this study still had a low level of cognition of CRC, and most of them did not participate in CRC screening. This significantly affected FDR’s compliance to early screening.

The FDRs included in this study were all family members accompanying patients with CRC during hospitalization, and they were involved in and witnessed the patient’s treatment. However, the level of cognition is still low. In clinical practice, doctors and nurses should strengthen health education for patients and their relatives and provide more detailed explanations of the patients’ diseases to increase the CRC cognition of patients and their FDRs. Additionally, the government and healthcare administration should also increase investment in medical resources and take more practical actions for disease publicity and prevention.

### 4.4 Inviting FDRs to participate in colonoscopy screening

After the FDRs completed and submitted the questionnaire, the doctor invited them to participate in colonoscopy screening and made a colonoscopy appointment for them. The results showed that the compliance of FDRs who received colonoscopy appointment sheets was significantly higher. Most of the FDRs in the group with colonoscopy appointments completed the screening in the hospital, which was significantly higher than that in the group without colonoscopy appointments. In addition, a subgroup analysis of FDRs who received colonoscopy appointments found that female, ≥ 40 years old, with higher family income, with a history of chronic diarrhea, and family members with multiple FDRs with CRC were more likely to accept the colonoscopy appointment, which may be the reason for the significant increase in colonoscopy screening among those who obtained colonoscopy appointments.

Our study provides an idea for improving compliance with colonoscopy screening for FDRs. Some researchers have taken various measures to enhance CRC screening compliance among FDRs, such as providing free face-to-face genetic counseling, inviting respondents to undergo colonoscopy screening using written materials, and conducting personalized risk screening assessments for FDRs (38–41). However, studies have shown that the main reasons for the rejection of colonoscopy screening by FDRs are fear of the trouble of colonoscopy, lack of time for colonoscopy, not knowing where to go for the examination, and fear of undergoing this examination (42). In this study, doctors actively invited FDRs to participate in colonoscopy screening and scheduled a colonoscopy appointment to fundamentally solve the problems encountered in the process of colonoscopy screening. On the one hand, the effectiveness of colonoscopy booking is long-term, and FDRs can avoid rejecting colonoscopy screening because of the short time. On the other hand, endoscopists could perform colonoscopy for the FDRs in this study in advance according to the green channel to avoid long waiting times and simplify the colonoscopy screening process. This colonoscopy appointment sheet was handwritten, and the subjects did not need to register and leave their identity information in the electronic medical record system. If the FDRs did not want to complete or did not have time for colonoscopy screening, there was no fee. In addition, no costs incurred and their credit was not affected, which greatly alleviated their concerns.

### 4.5 Limitations

However, there are some limitations in the study. First, this was a single-center preliminary clinical practice study. We only investigated colonoscopy screening compliance of FDRs and did not analyze the lesion detection. Second, the number of FDRs for the same CRC patient included in this study was limited, and the influence of family environment cannot be determined. Future studies can systematically screen for FDRs based on family units. And then, perhaps the age of onset of patients with CRC in the first family member is also a factor that affects the participation of FDRs in colonoscopy screening, but we were not able to further analyze the impact of this confounding factor on the findings. Finally, the investigators only issued colonoscopy appointments to FDRs who were willing to be screened, which may affect the reliability of the study results. A prospective randomized controlled trial (RCT) is underway at our center to reduce bias by randomly grouping FDRs through a rigorous screening program to confirm our findings in this study. In addition, more attention should be paid to the out-of-hospital FDR group after determining the effect on the improvement of screening compliance in FDRs during patient hospitalization.

## 5 Conclusion

This study found that being over 40 years old, having commercial insurance, having multiple family members with CRC, having a high level of cognition of CRC, and having high self-efficacy for disease screening were independent influencing factors for colonoscopy screening in FDRs. In clinical practice, inviting FDRs to undergo colonoscopy screening and issuing a colonoscopy appointment sheet may improve the compliance. Studies could further validate the feasibility of this approach in the future.

## Data Availability

The datasets presented in this study can be found in online repositories. The names of the repository/repositories and accession number(s) can be found in the article/**Supplementary Material**.
