# Supplementary material for: Influencing factors of colonoscopy screening in first-degree relatives of hospitalized colorectal cancer patients and preliminary clinical practices to improve the compliance
Source: Front Oncol. 2025 Apr 28;15:1533475. doi: 10.3389/fonc.2025.1533475 (PMC12066328; doi:10.3389/fonc.2025.1533475)
Supplement: Supplementary file 1 [file DataSheet1.docx]

| **Supplementary Table 1: Demographic characteristics and lifestyle of FDRs of patients with CRC (n = 303)** | | |
| --- | --- | --- |
| **Characteristics** | **n/mean** | **%/SD** |
| **Sex** |  |  |
| Male | 125 | 41.3 |
| Female | 178 | 58.7 |
| **Age(years)** | 42.82 | 11.43 |
| ＜40 | 126 | 41.9 |
| ≥ 40 | 177 | 58.1 |
| **BMI (kg/m^2^)** | 22.84 | 3.48 |
| **Employment status** |  |  |
| Part-time or without job | 142 | 46.7 |
| Full-time job | 161 | 53.3 |
| **Residence location** |  |  |
| Rural | 78 | 25.7 |
| Urban | 225 | 74.3 |
| **Marital status** |  |  |
| Single or divorced | 50 | 16.5 |
| Married | 253 | 83.5 |
| **Educational level** |  |  |
| Primary school | 23 | 7.6 |
| Junior high school | 85 | 28.1 |
| Senior high school | 72 | 23.8 |
| College/bachelor’s degree | 110 | 36.3 |
| Master degree or higher | 13 | 4.3 |
| **Family income (Chinese Yuan)** |  |  |
| < 2,000 | 58 | 19.1 |
| 2,000–4,000 | 83 | 27.4 |
| 4,001–6,000 | 109 | 36.0 |
| > 6000 | 53 | 17.5 |
| **Smoking** |  |  |
| No | 228 | 75.2 |
| Yes | 75 | 24.8 |
| **Alcohol consumption** |  |  |
| No | 247 | 82.5 |
| Yes | 53 | 17.5 |
| **Health insurance status** |  |  |
| Employee medical insurance | 112 | 37.0 |
| Resident or without medical insurance | 191 | 63.0 |
| **Commercial health insurance** |  |  |
| No | 200 | 66.0 |
| Yes | 103 | 34.0 |
| **Frequency of physical examination** |  |  |
| Never | 51 | 16.8 |
| Occasional | 160 | 52.8 |
| Regular | 92 | 30.4 |
| **History of chronic diarrhea** |  |  |
| No | 227 | 74.9 |
| Yes | 76 | 25.1 |
| **Willingness to free-finance screening** |  |  |
| No | 66 | 21.8 |
| Yes | 237 | 78.2 |
| **Family history of CRC** |  |  |
| 1 | 255 | 84.2 |
| > 1 | 58 | 15.8 |

FDRs: first-degree relatives; BMI: body mass index; CRC: colorectal cancer.

| **Supplementary Table 2: Compared with the demographic and lifestyle on FDRs with and without colonoscopy appointment sheet.** | | | |
| --- | --- | --- | --- |
|  | **Colonoscopy appointment sheet** | | **p value** |
|  | **YES (n=148)** | **NO (n=155)** |  |
| **Sex** |  |  | 0.019* |
| Male | 51(36.3%) | 74(44.2%) |  |
| Female | 97(63.7%) | 81(55.8%) |  |
| **Age(years)** |  |  | 0.002* |
| ＜40 | 47(32.7%) | 76(46.8%) |  |
| ≥ 40 | 101(67.3%) | 79(53.2%) |  |
| **BMI (kg/m^2^)** | 22.92±3.53 | 22.87±3.44 | 0.904 |
| **Employment status** |  |  | 0.706 |
| Part-time or without job | 71(49.6%) | 71(45.3%) |  |
| Full-time job | 77(50.4%) | 84(54.7%) |  |
| **Residence location** |  |  | 0.333 |
| Rural | 33(18.6%) | 42(30.0%) |  |
| Urban | 115(81.4) | 113(70.0%) |  |
| **Marital status** |  |  | 0.289 |
| Single or divorced | 21(15.9%) | 29(16.8%) |  |
| Married | 127(84.1%) | 126(83.2%) |  |
| **Educational level** |  |  | 0.137 |
| Primary school | 12(7.1%) | 11(7.9%) |  |
| Junior high school | 30(22.1%) | 49(31.6%) |  |
| Senior high school | 47(26.5%) | 28(22.1%) |  |
| College/bachelor’s degree | 53(39.8%) | 59(34.2%) |  |
| Graduate degree or higher | 6(4.4%) | 8(4.2%) |  |
| **Family income (yuan)** |  |  |  |
| < 2000 | 30(15.9%) | 33(21.1%) | 0.001* |
| 2000–4000 | 28(21.2%) | 55(31.1%) |  |
| 4001–6000 | 65(43.4%) | 39(31.6%) |  |
| > 6000 | 25(19.5%) | 28(16.3%) |  |
| **Smoking** |  |  | 0.664 |
| No | 113(72.6%) | 115(76.9%) |  |
| Yes | 35(27.4%) | 40(23.1%) |  |
| **Alcohol consumption** |  |  | 0.061 |
| No | 117(77.0%) | 135(84.2%) |  |
| Yes | 31(23.0%) | 20(15.8%) |  |
| **Health insurance status** |  |  | 0.866 |
| Employee medical insurance | 54(32.7%) | 58(39.5%) |  |
| Resident or without medical insurance | 94(67.3%) | 97(60.5%) |  |
| **Commercial health insurance** |  |  | 0.684 |
| No | 97(56.7%) | 105(71.6%) |  |
| Yes | 51(43.3%) | 50(28.4%) |  |
| **Frequency of physical examination** |  |  | 0.302 |
| Never | 31(14.2%) | 22(18.4%) |  |
| Occasional | 75(43.4%) | 85(58.4%) |  |
| Regular | 42(42.5%) | 48(23.2%) |  |
| **History of chronic diarrhea** |  |  | 0.007* |
| No | 106(68.1%) | 131(78.9%) |  |
| Yes | 42(31.9%) | 24(21.1%) |  |
| **Willingness to free-finance screening** |  |  | 0.558 |
| No | 26(13.3%) | 31(26.8%) |  |
| Yes | 122(86.7%) | 124(73.2%) |  |
| **Family history of CRC** |  |  | 0.003* |
| 1 | 113(76.1%) | 142(88.9%) |  |
| > 1 | 35(23.9%) | 13(11.1%) |  |

| **Supplementary Table 3: Effect of colonoscopy appointment sheet on FDR compliance with colonoscopy screening** | | | |
| --- | --- | --- | --- |
|  | **Colonoscopy appointment sheet** | | **p value** |
|  | **YES (n=148)** | **NO (n=155)** |  |
| **Colonoscopy screening** | 78(52.7%) | 35(22.6%) | <0.001* |
| **Screening in our hospital** | 45(30.4%) | 6(3.9%) | <0.001* |
| **Screening in other hospital** | 33(22.3%) | 29(18.7%) | 0.439 |

* p<0.05
